# Self-regulation and Beyond: Affect Regulation and the Infant–Caregiver Dyad

**DOI:** 10.3389/fpsyg.2016.00889

**Published:** 2016-06-15

**Authors:** Joona Taipale

**Affiliations:** Department of Social Sciences and Philosophy, University of JyväskyläJyväskylä, Finland

**Keywords:** infancy, interaction, pre-dyadic regulation, dyadic regulation, social referencing, differentiation, internalization, altero-matic regulation

## Abstract

In the available psychological literature, affect regulation is fundamentally considered in terms of self-regulation, and according to this standard picture, the contribution of other people in our affect regulation has been viewed in terms of socially assisted self-regulation. The present article challenges this standard picture. By focusing on affect regulation as it unfolds in early infancy, it will be argued that instead of being something original and fundamental, self-regulation developmentally emerges from the basis of a further type of affect regulation. While infants’ capacities in recognizing, understanding, and modifying their own affective states are initially immature and undeveloped, affect regulation is initially managed by the other: it is initially the self, and not the other, that plays the role of an assistant in affect regulation. To capture this phenomenon, the concepts of “auto-matic,” “hetero-matic,” and “altero-matic” affect regulation will be introduced and their interrelations elaborated. By showing how the capacity of affective self-regulation, which is characteristic to maturity, is developmentally achieved by internalizing regulative functions that, at the outset of development, are managed by the caregiver, it will be argued that altero-matic affect regulation is an autonomous type of affect regulation and the developmental basis for self-regulation.

## Introduction: Varieties of Affect Regulation

*Affect regulation* refers to the mechanism by which our emotions, moods, feelings, and their expressions are modulated in pursuit of an affective equilibrium or homeostasis. Its central relevance for the functioning of the human mind is being increasingly acknowledged in current scientific research and the topic has been approached from various perspectives. In the ongoing debate, the focal research interests and questions lie in the biological and evolutionary bases of affect regulation, in its cognitive underpinnings, in its development, in the correlation between regulative capacities and various personality features, and in various disorders of affect regulation as well as in their treatment. Characteristically, affect regulation is understood as “the process of initiating, maintaining, modulating, or changing the occurrence, intensity, or duration of internal feeling states and emotion-related physiological processes (…) often achieved through effortful management of attention (…) and cognitions that affect the interpretation of situations (…) as well as through neurophysiological processes” (Eisenberg et al., 2000, p. 137). The centrality of the phenomenon has been highlighted especially by Schore, who has argued that “the core of the self lies in patterns of affect regulation that integrate a sense of self across state transitions, thereby allowing for a continuity of inner experience” (Schore, 1994, p. 33).

In the vast majority of studies, “affects” are understood as intrapersonal or intrabodily states, and their regulation is portrayed in terms of *self-regulation*: regulation *of* the self *by* the self.^1^ According to this standard picture, our emotions and behavior are primarily regulated by our own attention, our own reactions, by our own cognitive acts and situational interpretations; in neurophysiological terms, the psychophysical state of our brain is modulated by our brain itself (e.g., Davidson et al., 2003; Perlman and Pelphrey, 2010; Siegel, 2012, pp 167ff.). Whenever *external* affect-regulative factors or ingredients (such as a soothing piece of music or the presence of a caring person) are considered, they are interpreted in terms of *mediators or facilitators of self-regulation.* That is to say, even if authors are today increasingly acknowledging the need to integrate a social perspective more intimately into the theory of affect regulation (e.g., Gianino and Tronick, 1988; Schore, 1994, pp 31–33; Totterdell et al., 1998; Parkinson et al., 2005; Niven et al., 2009; Beckes and Coan, 2011; Siegel, 2012; Butler and Randall, 2013; Fuchs and Koch, 2014), affect regulation is still fundamentally considered as a mechanism by which individuals modulate their own emotions, moods, and feelings (e.g., Gross, 2007). In short, the standard picture holds that, even if external factors may occasionally be *of help*, affect regulation nonetheless *fundamentally* amounts to regulation of the self by the self.

In the present article, I will be challenging this standard picture. Even if self-regulation undoubtedly has a relatively large role in the affect regulation – at least in adult life where we are held responsible for controlling our affective impulses and behavior – it might still be that not all affect regulation is in this sense literally “auto-matic,” i.e., set into motion or animated (Gr. *matos*) by something within oneself (Gr. *auto-*). To be sure, even “auto-matic” affect self-regulation may be passive or active (see Niven et al., 2009, pp 498ff.). On the one hand, it includes modifications that take place involuntarily, such as the performance-optimalizing functions of the autonomic nervous system. For instance, when we are mentally overloaded or feel stressed, the cortisol level in our body automatically increases, which paces up our heart rate and makes us more alert, thus making the situation (temporarily) more easily bearable; and when we are scared, our body “reacts” by increasing the amount of adrenaline in our circulation thus preventing us from collapsing or panicking. Experientially speaking, all this takes place unconsciously, meaning that we only experience the outcome. On the other hand, auto-matic affect regulation also includes more *active* modifications: when we sense a beginning headache, we might not just wait for our body or brain to take care of the problem by itself, but we may contribute to the regulatory process actively by stretching our tightened muscles, by closing our eyes and resting for a while, by trying to focus on something else than the pain (perhaps by meditation), or by taking painkillers, thereby medically erasing the nascent pain from within.

However, while trying to deal with an unpleasant feeling, besides modifying ourselves, we may also modify our environment. That is to say, the self may not only adjust to the prevalent external circumstances but also actively modify the latter to better match with one’s current affective moods and needs. For instance, feeling the nascent headache, we may dim the bright lights, put on some peaceful music, and so on, thus engaging in what has been called environmental “niche construction.” The term derives from the field of biology and refers to the manner in which organisms may actively transform their environment to make it fit with their needs – beavers building dams to enable an optimal ecosystem has been used as the paradigmatic example of such niche construction (Odling-Smee et al., 2003; Odling-Smee, 2009; Kendal et al., 2011). Just as beavers actively manipulate their inanimate surroundings, thus organizing their vital environment to their “pleasing,” as it were, we humans do so in various ways as well. Leaving untouched the question of niche construction *by human communities or human species* – a topic that would cover “culture” on the whole – even examples of *individual niche construction* alone appear innumerable. After all, here too one could discuss the whole scale of human needs: we *clothe* our body to regulate our body temperature, we establish a *dwelling place* to protect ourselves from environmental threats and changes in the weather, we dress up (in particular ways) to experience (particular kind of) social attention, we decorate our apartment to feel at home, we put on our favorite music to get into a particular mood before going to a party, and we switch the TV channel whenever the program feels uncomfortable for one reason or another. Despite the obvious motivational differences among these cases, all of them seem to share a common structure: they are all about *active manipulation of one’s immediate experiential environment in pursuing to modify one’s affective stance*. In contrast to what I called “*auto-*matic affect regulation” – where regulation is managed by something within *oneself* – I will call this “*hetero-*matic affect regulation,” since here the regulating source lies outside the boundaries of oneself (Gr. *heteros*: different, other-than). Hetero-matic regulation accordingly amounts to regulation of the self by something that is not the self.

This is not the whole story, however: there is still a further type of affect regulation, and the present article will be focusing on it. This form of regulation is *other*-based, and I will call it “*altero*-matic affect regulation” (Lat. *Alter*: another, second).^2^ What I have in mind here is the following. Besides the *aesthetic appearance* of others (i.e., the way they look, sound, and move) we are also affected by *their subjectivity* (i.e., their intentions, gestures, perceptions, reactions, moods, attitudes, emotional expressions, verbal reports, etc.), and we tend to be particularly sensitive when their experiences are *directed at us*. In everyday adult life, others function as “social mirrors” for us, and we tend to be rather sensitive in respect to what we see in the mirror (see Winnicott, 1971, pp 149ff.; Taipale, 2016b). Much of this “mirroring” takes place non-verbally: even the most discrete dynamic shifts in the other’s body language might betray appreciation, admiration, neutral acceptance, dismay, disagreement, blame, or overt ignorance (see Honneth, 2001, pp 122ff.), and the way in which we are perceived by others has a significant influence on how we feel. Standing out in a negative lighting is an unpleasant experience that we want to avoid; we care about our “social reputation” (Rochat, 2009, pp 118, 2), and the more so when it comes to people that personally matter to us. In this manner, others regulate our affective life. To a certain extent, just as with the environment, we can actively outline the nature of affective feedback we get from others. After all, by regulating what we do and do not let out, we at once *outline* the data that will be reflected back on us in the social mirror, and in this manner we may, to some extent, indirectly regulate our affective stance through others. Yet, our abilities in making others feel, think, or act in particular ways are rather limited, and – more importantly – other people also regulate our affective life without our initiative.

In this respect the case of *infants* is particularly interesting. As the infant’s self-regulatory capacities are initially immature and undeveloped, the affect-regulative role of the caregiver is emphasized. Yet, affect regulation has been studied in terms of self-regulation even when it comes to infants and toddlers (e.g., Calkins, 2007, pp 262–264; Hay and Cook, 2007, p. 119): the caregiver has been viewed as something that, besides the self, “also” modulates the infant’s affective stance (Thompson and Goodvin, 2007, p. 322). The caregiver has been explicitly treated as a mere “facilitator,” “helper,” or “mediator” in this allegedly self-centered process (e.g., Kopp, 1982, p. 200; Kopp, 1989, p. 345). Challenging this standard way of thinking, I will argue that in the very beginning the caregiver not only *adds* something to the infant’s own regulative efforts, but rather to a large extent *maintains* affect regulation on behalf of the infant: the caregiver is initially not just *in the service of* the infant’s affect regulation, but rather *in charge of* it. In other words, to a large extent, it is rather the self and not the other that initially plays the role of an assistant in affect regulation.

In what follows, I will engage in a developmental investigation and argue that auto-matic affect regulation arises from, and presupposes, altero-matic affect regulation. Challenging the standard view, in which altero-matic affect regulation is reduced to assisted self-regulation, I will suggest that self-regulation is gradually enabled along with the process in which the developing child increasingly internalizes a variety of regulative functions that are initially handled by the caregiver. Giving more support to my general claim concerning the fundamentality and autonomy of altero-matic affect regulation, I will argue that in the infant the latter tends to assume the form of hetero-matic regulation only when the care received by the infant is insufficient. Before concluding, I will illustrate the foundational relationship between altero-matic and auto-matic affect regulation from the point of view of psychopathology.

## Origins of Self-Regulation

The current research paradigm in developmental psychology builds largely on data that has been gained when infants are observed and examined during moments of so-called “alert inactivity”: the relatively brief periods when they are at their most cognitive, as it were. The concept of alert inactivity was originally coined by Wolff (1966), and later on made famous by Stern (1977, 1985). Moments of alert inactivity occur when an infant is neither sleeping, hungry, eating, fussing, crying nor engaged in full activity (Stern, 1985, pp 38–39). During such periods children perceptually explore their surroundings with neutral curiosity, almost like scientists. This, as Stern puts it, “provides the needed time ‘window’ in which questions can be put to newborns and answers can be discerned from their ongoing activity” (Stern, 1985, p. 39). While the existence of such periods or “time windows” is undeniable, and while it remains unquestionable that they take place fairly regularly – characteristically when the infant has just woken up and is not yet overwhelmed by needs or sedated by satisfactions – alert inactivity nonetheless is not the infant’s *prevalent* way of being, and by considering infants mainly during such moments therefore does not *comprehensively* capture the infant’s experiential world. As far as this is limitation is acknowledged, there is no problem; contemporary researchers are continuously discovering remarkable cognitive abilities in infants, and all these studies deserve to be greeted with praise. However, problems emerge if one “slides” from speaking of infants’ *experiential capacities and abilities* to speaking of their *experiential organization* more generally. In this respect, the formulations are indeed often too careless: instead of reading that “infants are *capable of differentiating* between self and other from birth” it is often said simply that “infants *differentiate* between self and other from birth.” Yet, to infer actuality from potentiality within a limited timeframe is a simple fallacy; the required conditions for the activation of the respective capacity (for differentiation) are not secured all the time – and not even most of the time. The mentioned “slide” is as justified as the claim that since human beings have the *faculty of reason* – after all, we are *animal rationale* – human life is all the time guided by reason. (We are not *that* reasonable.) Likewise, the fact that there *are* moments of “alert inactivity,” during which the various remarkable cognitive capacities of infants are actualized, is not a sufficient ground for assuming that the latter are operational all the time. Neutral curiosity is one, but not the dominant, original, or fundamental experiential mode of the infant^3^.

The reason for bring this up in the present context is that situations in which *affect regulation* is required are, by rule, situations in which the infant is *not* in the state of “alert inactivity.” When the infant is haunted by pressing needs, in plight, feeling uncomfortable, or sedated by overwhelming satisfactions, neutral curiosity remains *impossible* – not just *difficult* as in adult life – and the infant’s world is rather organized in the light of her felt needs and satisfactions. The distinctions fleetingly established, and documented, during cognitively oriented moments are not initially so deep rooted that they would, during the first weeks, go on to organize the infant’s experiential reality also during periods when the infant is hungry, for instance. In the baby, the sense of hunger is not present as a subjective topping to the objectively organized world. As Winnicott puts it, “being hungry is like being possessed by wolves” (Winnicott, 1964, p. 81): being initially incapable of seeing beyond the present moment, a hungry baby seems to behave as if it was the end of the world probably because she experiences hunger in such a manner – as there is nothing beyond the currently urging need, it seems to threaten her whole existence. Moreover, the hungry baby does not care about how the caregiver feels; she is not a good Kantian who treats the caregiver as “an end in itself.” The pressing need organizes the infant’s whole experiential reality, and the caregiver is initially nothing more than what she is in the light of the infant’s current needs and wants (Taipale, 2016a). Without taking any worth away from the magnificent findings that have been made when studying infants during periods of alert inactivity,^4^ what I am trying to convey here is this: in describing the infant’s experiential situation during affect regulation, we should not take for granted the experiential distinctions that are clearly manifest during moments of alert inactivity alone, when the infant is in the mode of neutral curiosity.

During periods when affect regulation is required differentiation between self and (m)other is all but clear, and it comes in degrees.^5^ Given that I am here focusing on altero-matic affect regulation, the main categorizing factor that I will be employing is the level of differentiation between *how one feels* and *how the other feels*. In the following, I will distinguish *three levels* in the developmental trajectory of affect regulation: “pre-dyadic regulation” (see section “Pre-dyadic Regulation”), “dyadic regulation” (see section “Dyadic Regulation: The Mirroring Other as a Beacon of Orientation”), and “increasing self-regulation” (see section “Social Referencing, Internalization, and Increasing Self-regulation”). These three levels, which surely also overlap, match with the threefold developmental division by Donald Winnicott, between “absolute dependency,” “relative dependency,” and autonomy or “independency” (Winnicott, 1965, p. 46).

### Pre-dyadic Regulation

At the very outset, there is what could be called “pre-dyadic regulation.” The infant does not yet distinguish between *how she herself feels* and *how the other feels*. To exemplify this, consider the phenomenon of *emotional contagion*. In a standard setting, when one baby in the room begins to cry, the other baby in the same room tends to follow suit. It would not be convincing to maintain that the second baby is just “empathizing” or “sympathizing” with the first baby, while maintaining a clear interpersonal distance. Unlike empathy and sympathy, emotional contagion is “self-centered” (De Vignemont, 2009, pp 63; cf. Scheler, 2008, pp 23ff.): the baby does not begin to cry because she experiences sadness *out there*, as it were, but because *she herself* feels sad. Neurophysiologically speaking, this is owing to the second baby’s functioning mirror resonance system (e.g., Gallese, 2009): the perceived sadness of the first baby *resonates* in the affective life of the second baby, so that the overt sadness, expressed in the cry of the first baby, is at once *felt* by the second baby in her own body. The emotion is thus “carried over” or “transferred” from one baby to another. What is at stake, however, is not a *cognitive confusion:* it would not be convincing to claim that the second baby begins to cry because she somehow believes or judges that the sad cry of the first baby is her own (this would not explain why the second baby, too, begins to cry). Rather than mistaking someone else’s sadness as her own, the infant senses an emotional presence and sucks in like a sponge. The feeling that the second baby is infected by is accordingly not felt by her *as* someone else’s feeling, but as her own (Scheler, 2008, pp 37, 15; Zahavi, 2015), and this is not confusion but an actual experience: the second baby actually *feels* sad. If the infant’s experience would be phrased as a question, it would *not* be in the active voice including several agents, “How do *I* feel in the presence of the *other*?” but rather more anonymously, in the passive voice, “How does *one* feel?”

Importantly, insofar as the experiential setting is not articulated in terms of a *dyad*, the infant is initially at the mercy of the sources of contagion. The feeling always already infects her, it resonates in her own body, before she “knows it” and critically asks: “Now whose feeling was this anyway?” As contagion is rather immediate, and there is no experiential distance to the influencing affect, the affective environment of the infant is uncritically taken in, and infants are initially strongly influenced by their affective surroundings: their feeling, tone, and mood, depends on the latter. As one can verify from everyday experience, babies tend to feel distressed when people around them are distressed, and they tend to feel calmer when people around them are calm; they catch the mood easily. The affective environment of the infant is multifarious and overwhelming, and if the sensory-affective external environment of the infant would not be organized by keeping an eye on her deficient affect-regulative capacities, the infant would be injured by the overwhelming stimuli. The infant’s self-regulatory capacities are initially immature and undeveloped, and if she is left to deal with demanding affective situations by herself, the outcome tends to be dramatic – e.g., in an overtly stressful environment a baby might withdraw psychologically and flee into apathy.

Here is where the caregiver comes in. In a certain sense, the infant’s primary caregiver is one source of affective contagion among others. However, the caregiver’s presence is very early on distinguished from the presence of *other* “others” – both *quantitatively* and *qualitatively*. On the one hand, the caregiver is the *prevalent* source: at the outset, she is present much more continuously than any other source, and the baby’s affective life is therefore mainly synchronized or attuned precisely with the caregiver’s affective life.^6^ On the other hand, the caregiver is not merely an affective *source*, an affective “contager” among others, but also something that has a special relationship with, an influence on, the rest of the infant’s environment: the caregiver regulates the infant’s exposition to other affective sources. To be sure, these sources might not be clearly differentiated from one another at the outset, but gradually, via repetition, this special influence or relationship *qualitatively* distinguishes the caregiver’s affective presence from the affective presence of other people.

To be sure, infants do have certain rudimentary means of affective *self-regulation*, such as instinctive turning away from overarousing stimuli (Gergely and Watson, 1996, p. 1186) and self-soothing thumb sucking. Yet, their capacities in affect regulation are rather insufficient at the outset, and neither are they capable of actively re-organizing their environment to their liking. When there is a bright light, the baby can close her own eyes, but when a noisy ambulance drives by, it is the caregiver that covers the baby’s ears. If everything came through, so to speak, the affective stimuli would easily be overwhelming. The maintenance of a “window of tolerance” in the infant’s affective life (Ogden et al., 2006, pp 26–40), a balance “between the accelerator and the brakes” (Siegel, 2012, pp 167, 315, 389), or “upper and lower limit control” (Bell, 1968, p. 88; Bell, 1971, p. 67)^7^ largely depends on the active role of the caregiver: what the infant cannot yet accomplish by herself, the caregiver is there to enable, and in this sense the latter serves as an undifferentiated segment in the infant’s relatively undeveloped “stimulus barrier” (cf. Kopp, 1989, p. 346).

Besides the negative, *affect-reductive* function, the caregiver also fulfills the infant’s basic needs, thus positively facilitating and managing the infant’s affective balance. Regulation undoubtedly begins already before birth; in several ways, the gestational mother regulates not just the infant’s biological functions but also the latter’s affective life – what she eats and drinks at once carries over to how the infant feels, singing and engaging in rhythmic movements is known to sooth the infant, and if the mother is distressed the cortisol of her blood also enters the circulation of the unborn baby. In this sense, there is a continuum, rather than break, between the infant’s pre-natal and post-natal life: the caregiver continues to regulate the infant’s affective life. In favorable circumstances, the newborn infant gradually *attaches* to the caregiver: she begins to favor, seek into, and desire for, this peculiar, increasingly differentiated experiential presence that, in her experience is, with increasing precision, associated with affective balance and well-being. However, even if *the caregiver’s affective presence* is increasingly differentiated from *the presence of other affective sources* – e.g., (the caregiver’s) protective hands vs. the disturbing noise (of the ambulance), – the former might not yet be clearly differentiated from *the infant herself*. In other words, for the infant, affect regulation is not experienced as *altero-matic*, other-initiated. Just consider the case of emotional contagion: what the infected baby is preoccupied with is the feeling, not the source of the feeling. During times of need, the caregiver plays the function of a regulative shield, and she might not be experienced as being anything more than that (Taipale, 2016a). The idea of there being, on the one hand, “feelings *in here*” and, on the other hand, “feelings *out there*” is not an issue at the outset of extrauterine development – rather, there are simply *feelings* that naturally resonate in the infant’s body.

What is said above can be summarized in terms of the distinction between other-*assisted* and other-*managed* affect regulation: the less developed the infant’s capacities of self-regulation, the more the caregiver not just *assists* but *manages* the regulation of the infant’s affects. The increasing share of other-*assisted* affect regulation in comparison to other-*managed* affect regulation is an indication of the infant’s increasing capacity of self-regulation – it marks a shift, in Winnicott’s words, from absolute to relative dependence. At the outset, the infant is “absolutely dependent” on the caregiver: given her remarkably insufficient abilities in self-regulation (Kopp, 1989, p. 345), without sufficient parental regulation she would succumb to the chaos of stimuli, she would be controlled *by* them, and consequently her development would become irreversively disturbed.

### Dyadic Regulation: The Mirroring Other as a Beacon of Orientation

A further factor promoting self/other differentiation relates to the fact that the caregiver’s affective presence is increasingly divided into such expressions that clearly *match*, or are *attuned*, with how the baby expressly feels, on the one hand, and to those expressions where such “contingency” is not detected.^8^ In other words, there is an increasing differentiation between “engaged” and “non-engaged” interaction (Reddy and Trevarthen, 2004). Gergely and Watson use the term “markedness” for the imitative contribution (or mirroring) within the caregiver’s expression by which she naturally signals that the infant’s present affective pattern is “seen” by the caregiver. Mirroring, echoing, or “attunement” is *amodal* in nature (Stern, 1985, pp 47ff.). What matters to the infant, what preoccupies her, is her present affective contour, and it is less relevant what sense region is emphasized as her feelings receive their expression. In other words, the infant naturally expresses her feelings with her whole body, and hence her *audible expression* – e.g., “oo-OO-*OO*-oo” – may be reflected back not only by a matching *sound-*pattern, but equally by a *visible* gesture that affectively matches the dynamic form of the infant’s original expression: in fact, perfect imitation of the original expression (i.e., “aping” it) may even be disturbing or irritating, aversive rather than inviting, and slightly different but affectively matching works the best.

In engaged or absorbed interaction, parental mirroring takes place without special effort, and it naturally invites the infant to actively continue the ongoing interaction.^9^ The infant reportedly tends to disengage when the caregiver no longer markedly mirrors the infant’s affects. This happens when the caregiver’s attention turns elsewhere – in the midst of interaction she may exchange a few words with her spouse, become lost in her thoughts, or start rambling with her smartphone. The break is decisive: as long as engagement is held up, the infant’s affective shifts and modes are echoed or mirrored in what she externally perceives – being surprised or delighted, for instance, is given immediate “feedback” in the form of a change in the affective presence of the caregiver. Once engagement is interrupted, how one feels no longer seems to have any effect in the environment. For the time being, contingency is gone; the caregiver’s face is no longer altered by the infant’s natural “invitations,” and she is thus prone to disengage: “If the mother’s face is unresponsive, then a mirror is a thing to be looked at but not to be looked into” (Winnicott, 1971, p. 152). As an extreme case of disengagement, just consider what happens in the “still face experiment” (see Tronick et al., 1975).

Via repetition, the infant begins to catch the qualitative difference between mirroring, attuned, and engaged affective data, on the one hand, and non-attuned or non-engaged affective data, on the other. The prominent distinguishing feature lies in what Gergely and Watson call “contingency detection” (Gergely and Watson, 1996, pp 1190ff.). The attuned affective data stands out as that which *matches* with how I feel; there seems to be a connection. In adult life, just consider feeling overtly sad and verbally telling someone about this; your experience will be remarkably different depending on whether the other person (i) becomes sad herself and starts to cry, (ii) whether in the other’s body language you clearly see that she realizes how sad you are (even if she would not “go along” with your sadness), (iii) whether she faces you with a neutral and unresponsive expression (saying mechanistically that she understands how you feel), or (iv) whether she does not seem to notice you at all. The first case is a matter of emotional contagion: you find the other being infected by your emotion and becoming sad herself. It is then *her own sadness* that she bodily expresses. By contrast, in the second case *your own* sadness is affectively mirrored back to you: unlike in the first case, what the other’s expression is here taken to convey is not the *other’s* feeling but your *own*: you find your own sadness receiving expression in the other’s body. In the third case, you may find your overt emotional state being considered from afar, perhaps evaluated or contemplated, but not mirrored back to you in the affective register; the others expression is not affected by your overt sadness: by maintaining a neutral expression, the other also maintains an interpersonal distance as if telling you, “*I* can see that *you* are sad.” In the last case, again, your emotion in not recognized at all: it is as if you were invisible to the other.

What interests us at this point is the second case. Unlike in the first case, what the other’s expression is here taken to convey is not the other’s feeling but your own. Differently put, *the other* would not look like this if *I* did not feel sad; it is *my sadness*, and *not the other’s sadness*, that makes her look like that, and in this sense the expression that I see seems to be “contingent” upon how I feel. Gergely and Watson argue that while, in early infancy, contingency is gradually and increasingly detected, how the infant herself feels and how the other feels are gradually differentiated or “decoupled” (Gergely and Watson, 1996, p. 1198). This discovery paves way to a significant developmental leap: realizing when it is her own affective life that is expressed in the other, the child gains and stabilizes a kind of *reflective stance* toward her affective life. To avoid confusion, it is not that what she finds in the other motivates her to engage in regular (intra-subjective) reflection, but rather that *by* looking at the mirroring other, the baby is *already* executing a kind of reflection: in engaged interaction with the mirroring other, she is gradually being *taught* how she herself feels.^10^ Such “teaching” assumes the form of what Gergely and Watson call “grouping”: various affective contours that the infant goes through in a non-articulated and pre-symbolic manner are, via consistent repetition, brought together in the expressive “feedback” of the mirroring caregiver. To simplify, the infant’s unpleasant affects (e.g., “UA1,” “UA2,” “UA3,” “UA4,” “UA5,” “UA6”), which are all expressed by crying, are increasingly grouped by the mirroring caregiver. As Bowlby puts it, “the ordinary sensitive mother is quickly attuned to her infant’s natural rhythms and, by attending to the details of his behavior, discovers what suits him and behaves accordingly” (Bowlby, 1988, p. 10). That is to say, “reading” her infant well, the caregiver’s mirroring response for “UA1,” “UA3,” and “UA4” differs from her mirroring response to “UA2,” “UA5,” and “UA6,” and hence, via repetition, the child gradually learns to categorize her unpleasant affective feelings into two kinds of unpleasant affective states (e.g., “UA1,” “UA3,” and “UA4” falling under “feeling of pain,” and “UA2,” “UA5,” and “UA6” under “feeling of hunger”). And gradually her expressions and actions begin to differentiate accordingly.^11^ The downside to this dependence is that if some of the child’s affects are not properly mirrored back – e.g., because they are considered forbidden by the parent – they will most likely remain unarticulated and ambiguous for the child as well, which is prone to give rise to difficulties in later life.

The capacity to dissect one’s affective life into clearly outlined categorical states is a later developmental achievement. Even if infants seem to naturally distinguish pleasurable affects from unpleasant ones, they hardly divide their affects into two clearly separate categories – and it would be even less convincing to claim that they initially categorize their unpleasant affective modes into states of “anger,” “hunger,” “pain,” “tiredness,” or “fear,” for instance. Before the infant learns to articulate and track (along with the above-mentioned “reflection”) her affective life in terms of specific affective “states,” her affective life already runs its course in the form of pre-reflective affective contours and tones – a phenomenon captured by the concept of “vitality affects.” The latter amount to the dynamic patterns of affective experiencing on the whole (Stern, 1985, pp 55ff.; Stern, 2010, pp 41–42), e.g., to the “crescendo” or “decrescendo” in bodily expression; they are *affective states in their pre-reflective (pre-grouped) givenness*.

Such indeterminacy initially characterizes the infant’s affective self-awareness, and the attuned caregiver amodally reflects back, and reacts to, their affective pattern or style, instead of considering them as fixed affective states. Gradually, the infant thus learns to thematize to objectify, affective dynamic patterns into particular “affective states.” That is to say, owing to parental mirroring, instead of simply feeling in a non-articulated manner, the infant gradually learns to track *how* she feels.^12^ And affect regulation crucially develops “in concert with children’s developing understanding of emotion and its meaning” (Thompson and Goodvin, 2007, p. 322). The parent is, in this fundamental sense, a “beacon of orientation” (see Mahler et al., 1975, p. 7): her mirroring expressions significantly teach the infant how she feels, thus serving as a guide in the midst of affective turmoil.

### Social Referencing, Internalization, and Increasing Self-regulation

The caregiver’s contribution to affect regulation at the outset of development can hardly be overestimated. At the outset, the caregiver largely *manages* the affective input of the baby, both by regulating his or her own affects and by actively steering (both negatively and positively) the infant’s exposure to other affective sources in the environment. As the dyad is increasingly formed and stabilized along with the differentiation between the caregiver and the environment, the infant begins to differentiate between expressions that reflect the caregiver’s own affective states, on the one hand, and marked expressions that mirror her own affective modes. This differentiation enables the infant to recognize, with increasing precision, her occurring feelings – to monitor them, to reflect upon their relationship with other affects, and hence to categorize and distinguish between them. This marks a decisive step in the development of the ability of self-regulation.

A further mirroring function of the caregiver is still to be discussed. As the infant becomes more skilled in distinguishing the caregiver’s mirroring expressions from expressions that reflect the caregiver’s own feelings, she also becomes increasingly sensitive to the *way* in which her affects are mirrored. The infant gradually learns to differentiate between two modes of mirroring: those signaling situational approval and those signaling situational disapproval. Along with increasing differentiation in this respect, the parent gains a *normative* significance: the caregiver’s face indicates not just how one feels, but also whether how one *should* presently feel (i.e., whether or not this spontaneous feeling or reaction is appreciated in current circumstances). In other words, the caregiver is a “beacon of orientation” not just in the *descriptive* but also in the *normative* sense: besides consulting the caregiver while seeking an answer to the question, “How do I feel?,” the infant now increasingly seeks an answer to the question, “How should I feel?”

This issue has been discussed under the rubric of “social referencing” (e.g., Feinman, 1992). Social referencing refers to the act of assessing the reactions of significant others and seeking guidance in them, in order to determine how to act, think, or feel in a particular situation. Unlike in adult social life, where the phenomenon is also widely operative, in the very first forms of social referencing – emerging usually by the end of the first year (Gergely and Watson, 1996, p. 1187) – the experience tends to be much more *suggestive*. Consider the case in which a 9-month-old baby is about to take a step without support, and just before doing so turns to consult the caregiver’s facial expression. In the words of Gergely and Watson, the infant might be “actively seeking out a clarifying affect-mirroring cue from the parent that will result in the strengthening and coming to dominance of one of the conflicting emotion-states he/she is currently in, thereby resolving his/her indecision” (*ibid*.). The infant clearly *wants* to take a non-supported step but feels afraid in the face of the uncertain outcome, and to resolve her affective indecision, she consults the parent’s face – not in order to know *how she herself already feels* (or to know *how the caregiver feels*), but to know *how she herself should feel*. If the adult looks uncertain and afraid, the infant tends to give up the idea, and if the parent is overtly supporting, smiling and “giving a green light,” the infant will more easily try taking an unsupported step. What the parent’s expression here regulates is the infant’s affective stance (and, therefore, her action as well). Likewise, whenever the infant harmlessly falls and bumps herself to the floor after a failed first try, she is prone to consult the parent – and, again, if the latter stays calm (perhaps making a markedly funny face and saying “whoops!”) the child also will much more likely feel calm about what happened and reacts less dramatically than when the parent is overtly terrified and concerned. Besides future-oriented suggestions, social referencing is also used to evaluate something that has already taken place; instead of consulting the caregiver, asking, “Is this OK?,” the infant also consults the caregiver, asking, “Was this OK?” Consider that at a dinner table a toddler deliberately pours a full glass of milk onto the table, cheerfully smiles and titters, and then carefully turns toward the caregiver (as if for consultation, “How about that?”). The affective impact upon the child is different depending on whether the caregiver just smiles and laughs along, thus signaling *approval*, or whether she recognizes the infant’s cheerfulness, but holds a more serious look, thus signaling that deliberately messing up with food is not a laughing matter.

Now, along with the increasing capacity to track how she feels, the infant begins to anticipate dyadic mirroring of her affective experiences: parental mirroring is gradually *internalized*. Consider an infant who is learning to walk, and who, each day, falls dozens of times. Consider that the infant does not hurt herself that badly, and that her caregiver greets her efforts with soothing encouragement (“It’s ok, just try again”). After a while, when the infant falls, she already knows to expect such soothing encouragement, and her affective reaction to falling down is, in this sense, less dependent upon actual parental consultation – she no longer each time seeks the gaze of the parent and, at some point, does not any longer require the caregiver’s actual presence to feel the soothing encouragement: “It’s ok, just try again.” Negative situational mirroring patterns become likewise internalized. Consider the milk-pouring example: by being consistently mirrored with a negative tone each time she deliberately pours her milk glass, the child gradually internalizes the situation-dependent blame, and is able to anticipate the unpleasant outcome. In this manner the child becomes learned in what could be called *preventive affect regulation*: avoiding situations usually leading to unpleasant other-based affects (such as blame or shame).

Internalization of affect mirroring enables a leap in the child’s capacities in affective *self-regulation*: the child increasingly follows social expectations also in the absence of external monitors, and she leans on the internalized presence of the altero-matic regulator also when the latter (the caregiver) is not actually present (cf. Kopp, 1982, p. 206; Kopp, 1989, p. 350).^13^ Consider the behavior of a child that is put to sleep. During the first months of life, when the continual presence of the caregiver is needed, the infant easily begins to cry when she is laid down and left alone. Gradually, however, she cries less and less in such situations, and at a certain age, instead of crying, thus actively calling the actual parent to sooth her, the infant increasingly learns to keep herself company, as it were – e.g., she “babbles” or “chatters” by herself, she holds on to and hugs her familiar soft toy, etc. This capacity to be alone for a while marks an important developmental achievement: the presence of the actual caregiver in flesh and blood is no longer continually required, because the infant has internalized her and carries her with her, as it were. When the caregiver is not actually present, the infant is able to endure the felt absence by *playfully invoking* her presence. This is not a matter of hallucination or memory: the child does not posit, actually believe in, the real existence of the caregiver (e.g., if the caregiver actually re-enters the room, the child hardly experiences this as the arrival of a second caregiver), whereas on the other hand she does not refute the caregiver’s existence either (in this case, the “created” presence would hardly be soothing). For an analogical experience in adult life, just consider watching a thriller movie: when you are absorbed in the film, you might feel afraid when in the film a murderer suddenly jumps from the closet, but when that happens you do not normally call the police (i.e., watching a movie you do not truly judge that there is actually a murderer in front of you); on the other hand, if you treat the thriller as a being only a movie, and judge the murderer not actually being there, you would not be afraid and could not enjoy the movie. Likewise, the infant playfully, spontaneously, *re-enacts* the caregiver’s presence, without making existential judgments one way or the other, as it were – questions of actuality remain undecided (Winnicott, 1971, pp 17–18; Scheler, 2008, p. 24).

Insofar as the infant carries the primary caregiver within her, she is increasingly capable of affect regulation also outside the infant–caregiver dyad. Gradual internalization thus slowly facilitates the infant’s process of separation from the primary caregiver, thus preparing a way toward independence (also) with respect to affect regulation. Gradually differentiating herself from the primary caregiver enables further dyadic relationships: other people, too, are constituted as social mirrors, and different people reflect the child’s affective states differently. Increasingly differentiating between these alternative self-evaluations, she also begins to favor some over others. For example, knowing that her mom is stricter about cookies, the child tends to ask her dad whether she could have one more – her dad is expected to mirror her desire in a more approving lighting. More generally, as the child gains more reflective surfaces to her feelings and impulses, alternative perspectives to the appropriateness and inappropriateness of her affective expressions, her social self-awareness and situational prudence become gradually structured. By entering the social world, the child no longer *uncritically* takes the caregiver as *the norm* of how one should feel in such-and-such circumstances, and in this sense she grows more independent: by internalizing alternative social mirrors, as it were, she also learns to disagree with the primary mirror. To know how she is expected to feel, the child no longer exclusively consults the (actual or internalized) *caregiver*; her question is rather more social and general: “How am I expected to feel *by these-and-these others*, and how do *I myself* feel?”

## Altero-Matic Regulation: Originality, Autonomy, and Pathology

We are now in the position to systematically examine the relationship of altero-matic regulation to auto-matic and hetero-matic regulation, and argue that it is a *sui generis* form of regulation. The basic reason why altero-matic regulation cannot be reduced to assisted self-regulation is that in early infancy the child’s subjective regulative capacities are remarkably insufficient. To think that all that the parent does is to support the infant’s self-regulation is as reasonable as saying that a parent “supports” a teenager in cleaning her own room by cleaning it herself. Infants are born helpless: they enter the world in a state of *absolute dependency* (Winnicott, 1965, p. 46), meaning that the caregiver is initially there not just to *assist* the infant’s auto-matic regulatory processes, but to *manage* them (Kopp, 1989, p. 345). The infant’s dependency on the caregiver gradually decreases as she increasingly internalizes the regulative functions of the caregiver. Emerging independence in this respect enables the infant to begin to “make use of” other people for self-regulative purposes. That is to say, what I hope to have shown above is that whenever altero-matic regulation is considered in terms of assisted self-regulation (as it mostly is in the literature), a long and complex developmental process is already presupposed: a process in which the subject is at first dependent on the regulating presence of the caregiver. In short, auto-matic affect regulation *is founded upon* altero-matic affect regulation, and the latter hence cannot be reduced to the former.

Partly for the same reasons, altero-matic regulation cannot be reduced to hetero-matic regulation. For one, the infant is initially unskilled in body-control, and she herself is at first simply unable to adjust the environment to fit her moods and needs.^14^ Yet, even as the child develops, enters into adulthood, and becomes capable of “using” the environment to regulate her affects, this, by rule, requires intentional activity: the environment does not adjust to one’s moods and needs *by itself*, even if it does not in itself *resist* active manipulation either. In this respect, altero-matic regulation structurally differs from hetero-matic regulation in two senses: the other is not just passive but active and might hence both (1) *resist* our attempts of manipulation and (2) *regulate our affects without any intentional activity on our part*. The first phenomenon is familiar from our everyday social life, where the gap between our own affective life and the affective life of others is relative clear: we might *ask* the other to do something, but the outcome is, in principle, unsure. By contrast, a stereo equipment does not have plans of its own; when we want to hear our favorite song, we can just put it on. The second phenomenon is likewise characteristic to everyday social life, though more emphatic in infantile experience. The stereo equipment does not track our emotional states, and function accordingly without any intentional activity on our part, but the other person might see that we are sad, for instance, invite us to talk about it, or put on our favorite song, thus in various ways “mirroring” our feelings without asking for our permission. That is to say, without any active effort on our part, others may regulate our affects, and they may do this either directly or by modifying our environment. In certain circumstances, however, significant other persons may themselves be present as figures within the environment. In a favorable developmental setting, care can be taken for granted (Taipale, 2016a): the infant does not usually have to beg and beg for the caregiver to react to her needs and wants; from the most discrete signals the “primarily preoccupied” caregiver (Winnicott, 1984, pp 300ff.) often recognizes her infant’s needs already before the infant herself knows what is it that is beginning to bother her – and acts accordingly. In good cases, then, care arrives without active effort and the caregiver is not present as someone that has to be manipulated in order to achieve affective relief. Care comes without “asking,” and the self is rather passive vis-à-vis the affect-soothing object. If, by contrast, care is not sufficiently continuous and consistent – i.e., not “good-enough” – the infant is forced trying to actively influence the caregiver, and while partly failing to do so, the care appears to be something that one cannot take for granted – this promotes early separation which has harmful consequences to the subsequent emotional development.

The affect-regulative effect of the affectively mirroring other is reportedly *qualitatively* different from affect regulation by a non-responsive object – even when the latter, too, is a living being. In a now classical “double video” study, Murray and Trevarthen (1985) reported that already in their third month infants are able to discriminate between a live video of their mother (who at the same time sees the infant in another screen) from a recorded videotape (cf. Nadel et al., 1999). Using similar settings, several researchers have argued that infants are significantly more interested in their mother during a live feedback than during a replay sequence (e.g., Muir and Nadel, 1998, pp 256–259; Nadel and Tremblay-Leveau, 1999, pp 195–200; Stormark and Braarud, 2004). Such studies suggest that engaged, mirroring presence significantly differs from non-mirroring presence very early on; when the caregiver disengages, the infant tends to disengage too. This nicely fits with what has been said above, namely that whenever the other is not engaged with oneself, in her affective presence she moves into the background, becomes part of the affective environment, that has to be actively manipulated to have the desired affective impact. To apply and generalize this idea: listening to an audiobook and listening to someone reading a book to you has a different effect, and to “baby-sit” a child by making her watch a television program is not quite the same as live interpersonal interaction (even if extremely capturing). In live interaction, the storyteller adjusts to the child’s expressions, gestures, and reactions, aligns and attunes with the reception of the fairytale; a cartoon, by contrast, does not care about how this particular child feels while watching it. The decisive feature distinguishing altero-matic and hetero-matic affect regulation is accordingly not in the distinction animate/inanimate but in the distinction between mirroring and non-mirroring objects. Whenever an object does not mirror one’s affective states, it is not present as an interacting other, but part of one’s affective surroundings.

One further distinguishing feature is worth highlighting as it supports the claim of the *sui generis* nature of altero-matic affect regulation: in the latter, *the actual presence of the regulator* is not necessary. Once the presence of the other is internalized, it continues to play its affect-regulative role also when the other is absent. In the case of auto-matic and hetero-matic regulation, the presence of the regulator is required. My headache is not altered simply by the *possibility of* stretching, by the *possibility* of taking a painkiller, nor is my stress relieved simply by the possibility of listening to some Bach or Haydn, or merely thinking about my favorite song^15^, but the sheer *possibility* or *thought* that others (or a particular other) may see me stealing something, for instance, even if I am sure they presently do not, may give rise to enormous guilt. In fact, some people that are (presently or permanently) absent may even have a *stronger* influence on the organization of our affective life than those that are actually present at the moment.

Yet, even if the parent is gradually “internalized,” and becomes, in a way, one social mirror among many, the (internalized) caregiver is not just *a* mirror, to rephrase Winnicott, but also the “precursor” of the mirror, and the way that one is mirrored in early childhood largely colors the way one expects oneself to be viewed later on (Winnicott, 1971, p. 149). Given the developmental roots of auto-matic affect regulation in internalized altero-matic affect regulation, what the infant internalizes in the earliest developmental stages is destined to largely outline her capacities in affective self-regulation. Favorable development is not a given here: early interaction and the type of attachment in a way sketch a developmental direction, even if not a determinate “fate,” for subsequent self-regulation.^16^ I have up until now presupposed that the care received by the infant is *good-enough*. If, by contrast, the child is not mirrored properly, or is mirrored ambiguously or mainly negatively, a lot is at stake: along with the insufficient capacity to learn to control one’s own affect, the whole emotional development of the child is distorted. Ainsworth et al. (1978), Bowlby (1988, pp 140ff.), Main et al. (2005), distinguish various “attachment patterns” in early development, each of which offers a particular kind of basis for the subsequent development of self-regulation: (1) *secure*, (2) *avoidant*, (3) *resistant* or *ambivalent*, and (4) *disorganized* or *disoriented* attachment (cf. Siegel, 2012, p. 97ff.). The division is established from the basis of the so-called “strange situation test,” which measures the child’s capacity to adjust to new circumstances when the parent leaves the scene. In our terms, then, the test tracks precisely *the developmental stage of self-regulation in the absence of altero-matic regulation*.

In *secure attachment* (or, as we could also say, in secure altero-matic affect regulation), the caregiver is attuned to the infant’s affective life. Holding attachment in value, the caregiver is emotionally available, perceptive and responsive to his or her infant. In such a setting, the infant is consistently perceived, understood, and responded to by the caregiver (Bowlby, 1988, p. 140; Ogden et al., 2006, pp 47ff.; cf. Siegel, 2012, pp 99–101). The child’s “beacon of orientation” is a stable, consistent, and reliable standpoint, and as she gradually internalizes the former, her capacities in self-regulation are provided with optimal developmental conditions, insofar as the infant does not have to compensate the lacking altero-matic regulation with immature and hence dramatic self-regulative efforts. By contrast, whenever altero-matic affect regulation has been remarkably insufficient one way or the other, problems tend to ensue. If the altero-matic regulator is not reliable or consistent, for instance, to maintain her emotional balance the child’s own regulatory efforts come to compensate or complement the respective insufficiency.

In *avoidant attachment*, the parent is incoherent, emotionally unavailable, imperceptive, unresponsive, and dismissive, and the infant’s altero-matic affect regulation is thus insufficient: her affective states are not properly mirrored or reflected in the parent, and the child usually reacts by less easily externally revealing how she feels.^17^ Interestingly, Main et al. (2005, esp. 275–276) have reported that while both securely and avoidantly attached infants were later on equally capable of *categorizing* their negative feelings, the avoidantly attached children were less skilled in *dealing with* the negative emotion in question. In the avoidant attachment pattern, the regulation of external expression is heightened: to avoid shame arising from the (repeated) situation in which one spontaneously expresses one’s feelings but is not seen (or worse: is humiliated), the child tends to become rather cautious in respect to what she externally reveals, and this complementary deliberation may assume the form of what Winnicott calls a narcissistic false self organization (Winnicott, 1965, pp 140ff.; Bowlby, 1988, p. 140).

In *ambivalent attachment*, the parent is inconsistently and unpredictably available, sensitive, perceptive, and responsive (Bowlby, 1988, p. 140; Ogden et al., 2006, p. 50). The parent might try to bond with the child, but in a way that is not contingent to the child’s communication but to the parent’s (see Siegel, 2012, p. 128). The parent is prevalently present in an ambivalent manner, neither consistently engaged nor consistently disengaged.^18^ In classical psychoanalytic terms, in secure attachment the parent enables an extensive period of omnipotence for the infant, whereby the infant’s affective life is organized as something that has a social, real, impact in the world; in avoidant attachment, the unresponsive parent remains in the background, and the infant tends to become separated rather early on, before the sense of her social relevance is properly established; and in ambivalent attachment the child has grown in a setting in which she has not been able to be sure whether her feelings will be seen, acknowledged, overlooked, or rejected. As compensation, the child’s attachment system becomes “overactivated,” as it were: the child is hungry for interaction and is desperately seeking it, even when the parent is engaged, as if to ensure the continuation of engagement. To internalize such a “beacon” tends to entail imbalanced emotional development: if the “precursor of the mirror,” the primary regulator, is ambiguous, this ambiguity will be, along with internalization, carried over to the self.^19^ Basic sense of insecurity is compensated with a desperate, insatiable search for security.

Finally, *disorganized attachment* is given rise when the child grows up in an intensively frightening environment (Main and Solomon, 1990): she is continuously physically abused, mistreated, or neglected, or mirrors herself in a parent who is herself caught up with an unresolved trauma or object-loss which has left him or her drifting aloof from the surrounding reality or made her psychotic. The horror of the situation lies in the fact that, even if the child fears for her life, she has nowhere else to flee but toward the source of her distress. In the unbearable situation, where actual escape is not possible, the child has no other means but to flee mentally and detach herself from what’s really happening. If the condition is continuous, a rule rather than an exception, the possibility of later dissociative disorders and psychoses increases: the mind of the child threatens to split into various disconnected realms (see van der Hart et al., 2006, pp 6–7; Liotti, 1992). In short, a disorienting “beacon” is really not a beacon at all, and to internalize something like that is to be at a loss.

In this manner, developmental psychopathology can illuminate the relation of dependence between auto-matic and altero-matic regulation, and hence also highlight the difference between them. Altero-matic regulation cannot be reduced to an assisted form of auto-matic regulation. By contrast, hetero-matic regulation can sometimes be considered as a mediated form of self-regulation: environment does not adjust itself to my needs; intentional activity is required for this. When I put on music to get into a particular mood, I initiate the affective modification even if the environment functions as the primarily affective source. However, as has been said above, my environment can be manipulated also by other people. In such cases, hetero-matic regulation would not be reducible to (an indirect form of) auto-matic regulation, but to (an indirect form of) altero-matic regulation. Given the complexity of everyday social life, all these forms, along with their indirect variants, have a share in our affective life, and it may be hard to distinguish them from one another. Yet, given the developmental primacy of altero-matic regulation, and the fact that auto-matic regulation is largely derived from the latter, we can maintain that altero-matic regulation is an original phenomenon.

## Conclusion

I have here suggested that the standard picture, according to which affect regulation is interpreted in terms of (assisted) self-regulation is misleading, because the latter is developmentally based on another type of regulation: the manner in which we are capable of regulating our affects – whether directly or by modifying the environment – largely depends on the developmental course of what I have called *altero-matic affect regulation*. Venturing through pre-dyadic and dyadic forms of early affect regulation, I showed how self-regulation becomes increasingly independent as the developing child gradually internalizes the regulative functions that are initially managed by the caregiver. In this manner, self-regulation is gradually established from the basis of altero-matic affect regulation, and altero-matic regulation therefore cannot be reduced to assisted self-regulation. Self-regulation becomes possible thanks to a particular kind of altero-matic affect regulation, which makes it understandable that if something goes wrong in the latter, the traces of this are to be found, even if only later on, in the former.

## Author Contributions

The author confirms being the sole contributor of this work and approved it for publication.

## Conflict of Interest Statement

The author declares that the research was conducted in the absence of any commercial or financial relationships that could be construed as a potential conflict of interest.

## Footnotes

1 Self-regulation is sometimes divided into attention regulation, emotion regulation, and behavioral self-control (including the regulation of bodily expression; e.g., Forman, 2007, pp 294, 321; Siegel, 2012, pp 299ff.). It is generally thought that regulation does not change the quality or “valence” of a given affect (e.g., a *painful sensation* does not turn into a *pleasant sensation*), but rather influences the “dynamics,” one’s “take” on, the affective experience in question (see Forman, 2007, p. 321): it consists in monitoring, evaluating, and modifying affectivity, especially its “intensive and temporal features” (Thompson, 1994, pp 27–28), quantity, frequency, intensity, escalation, and emotional response (Thompson, 1990). For a discussion on the functional aims of self-regulation, see, e.g., Russell and Feldman Barrett (1999), Feldman Barrett et al. (2001), Campos et al. (2004), and Gross (2008). For an extensive overview of the concept of self-regulation in recent literature, see Burman et al. (2015).

2 Bråten (2007) has coined the concept of “altercentric participation,” which is related, but not identical with the concept I am introducing here.

3 Concerning experiments speaking for the various, remarkable, extroverted capacities of infants (e.g., experiments of neonatal imitation, still-face experiments, etc.; e.g., Dondi et al., 1999; Farroni et al., 2004; Rochat, 2004; Nagy, 2008; Reddy, 2008, pp 120–149).

4 Elsewhere, I have argued that theories advocating early differentiation and theories advocating early undifferentiation should be seen as complementary to one another (Taipale, 2013).

5 See Taipale (2016a).

6 Trevarthen and Aitken (2001) report: “Infants only 2–3-months old move their arms in synchrony with their caregiver’s talking rhythm and create synchronous way of relating to the caregiver.

7 As Bell explains, the caregiver’s “upper limit control” aims at modifying the behavior of the infant, whereas “lower limit control” is directed at stimuli itself: “In other words, the parent’s control behavior, in a way, is homeostatic relative to child behavior” (Bell, 1968, p. 88).

8 Detection of social contingency has recently been reported already in 2-month-old infants (Soussignan et al., 2006).

9 See Rochat (2009, pp 67ff.) on the emergence of “co-awareness” at the age of 6 weeks.

10 This reflecting or “monitoring” is crucial in the development of regulative capacities: “Emotion regulation emerges in concert with children’s developing understanding of emotion and its meaning” (Thompson and Goodvin, 2007, p. 322). Moreover, along with this, the infant learns to increasingly *co-regulate* her affects with the caregiver (Gianino and Tronick, 1988; Beebe and Lachmann, 1998).

11 Fonagy and Target (2005, p. 339) discuss this phenomenon in terms of the caregiver’s “reflective function,” and claim that “mothers with high reflective function possess greater capacity to regulate the baby’s fear, interacting with her without frightening or otherwise disorganizing the baby.”

12 For an analogy in adult life, consider looking at a mirror and realizing that you *look* tired; consider, moreover, that you had not explicitly categorized your feeling as “tiredness,” even if you felt tired already; now, looking at a mirror, you see how tired you *look*, and this makes you realize that you indeed *are* tired.

13 Consequently, the child may feel shame over something that she does in privacy or even over her mere thoughts: a sheer idea of others perceiving us suffices.

14 As is noted by Derryberry and Rothbart (2001), at the outset, intrasubjective affect regulation is guided primarily by intrinsic (neuro)physiological factors.

15 To be sure, I can be taking a placebo painkiller for my headache, and that might in some cases, make me feel better, but this does not work if I simply imagine, recollect, or fantasize about taking a painkiller.

16 On the retrospective significance of early development of affect regulation, see also Thompson and Goodvin (2007, pp 324ff.; cf. Noller, 2005).

17 In terms of Hill-Sonderlund et al. (2008, pp 262ff.), the child is thus carrying a greater “allostatic load” (Hill-Soderlund et al., 2008). On the imbalance between the child’s nervous system responses (measured by heart rate variability) and her external behavior, see Fox and Hane (2008, pp 222ff.) and Siegel (2012, pp 120ff.).

18 Often, there are two primary caregivers, and if they respond to the child in a very different manner, their presence may be ambivalent when considered as unity. Insofar as the emotional responses of the parents are not too divergent from one another, the infant’s affective self-presence is organized more or less coherently.

19 Insufficient self-regulation has also been linked with hyperactivity and inability to inhibit inappropriate behavior (Forman, 2007, p. 294).
